# A Head-Mounted Multi-Camera System for Electrophysiology and Behavior in Freely-Moving Mice

**DOI:** 10.3389/fnins.2020.592417

**Published:** 2021-01-18

**Authors:** Nicholas J. Sattler, Michael Wehr

**Affiliations:** ^1^Department of Biology, Institute of Neuroscience, University of Oregon, Eugene, OR, United States; ^2^Department of Psychology, Institute of Neuroscience, University of Oregon, Eugene, OR, United States

**Keywords:** natural behavior, active sensing, freely-behaving animals, cyclotorsion, retinotopic, head-mounted camera, eye movements

## Abstract

Advances in the ability to monitor freely-moving mice may prove valuable for the study of behavior and its neural correlates. Here we present a head-mounted multi-camera system comprised of inexpensive miniature analog camera modules, and illustrate its use for investigating natural behaviors such as prey capture, courtship, sleep, jumping, and exploration. With a four-camera headset, monitoring the eyes, ears, whiskers, rhinarium, and binocular visual field can all be achieved simultaneously with high-density electrophysiology. With appropriate focus and positioning, all eye movements can be captured, including cyclotorsion. For studies of vision and eye movements, cyclotorsion provides the final degree of freedom required to reconstruct the visual scene in retinotopic coordinates or to investigate the vestibulo-ocular reflex in mice. Altogether, this system allows for comprehensive measurement of freely-moving mouse behavior, enabling a more holistic, and multimodal approach to investigate ethological behaviors and other processes of active perception.

## Introduction

Mice move. Whiskers whisk, eyes saccade, nostrils sniff, and ears pivot (Preyer, 1882; Welker, 1964; Carvell et al., 1991; Wallace et al., 2013). These active sensing behaviors have been well-established as adaptive strategies for the optimization of somatosensation (Gibson, 1962; Lederman and Klatzky, 1987; Carvell and Simons, 1995; Bagdasarian et al., 2013), vision (Ballard, 1991; O'Regan and Noë, 2001), olfaction (Mainland and Sobel, 2006), and audition (Populin and Yin, 1998; Holland and Waters, 2005; Tollin et al., 2009). Recent studies have also revealed that movement has widespread neural correlates throughout the brain. Eye movements, whisking, sniffing, running, and other classes of movements can each produce independent effects on brain regions far removed from the directly-involved motor or sensory systems (Musall et al., 2019; Stringer et al., 2019; Salkoff et al., 2020). Investigating sensory processing therefore requires either that all movements are eliminated (e.g., by head or eye fixation), or that they are accurately measured. Despite the insights that have been gained from research under head-fixed or eye-fixed conditions, there has been a growing appreciation that understanding brain function will require recording neural activity in freely-moving animals, especially during ethological behaviors (Datta et al., 2019; Parker et al., 2020). Yet monitoring the simultaneous movements of the eyes, whiskers, nose, ears, limbs, and the rest of the body in freely-moving conditions and with high-density electrophysiology remains methodologically challenging.

There are many inherent technical difficulties when designing attached devices for freely-moving behaviors in small animals. Every component comes with an associated cost of size, weight, positioning, balance, and cabling. Each of these factors necessarily constrains the free movement of the animal, and the magnitude of their effects scale inversely with the animal's size and strength. The first rodent head-mounted camera system was developed to monitor the behavior of freely-moving rats, with each attached camera weighing 800 mg (Wallace et al., 2013). A more recent breakthrough was the development of a head-mounted system for monitoring both behavior and electrophysiology in mice, with each attached camera weighing 500 mg (Meyer et al., 2018). Mice are an order of magnitude smaller than rats however, therefore requiring significantly smaller and lighter head-mounted devices. Here we describe a promising new system for monitoring physiology and behavior in freely moving mice, with significant advantages in size, weight, cabling, signal quality, and customizability. The key advance is the use of miniature analog camera modules (180 mg, 5 × 5 × 5 mm), allowing for lightweight multi-camera headsets, finely-focused video signals, and single-ended signal connections for low-impact tethering.

We present two examples of multi-camera headsets for use with electrophysiology: a headstage-integrated four-camera headset, and an adjustable two-camera headset. We then provide further examples of their implementation, and discuss general guidelines for designing a specific implementation of this system to best suit your experimental goals.

## Results

### Four-Camera Headset

**Figure 1:**
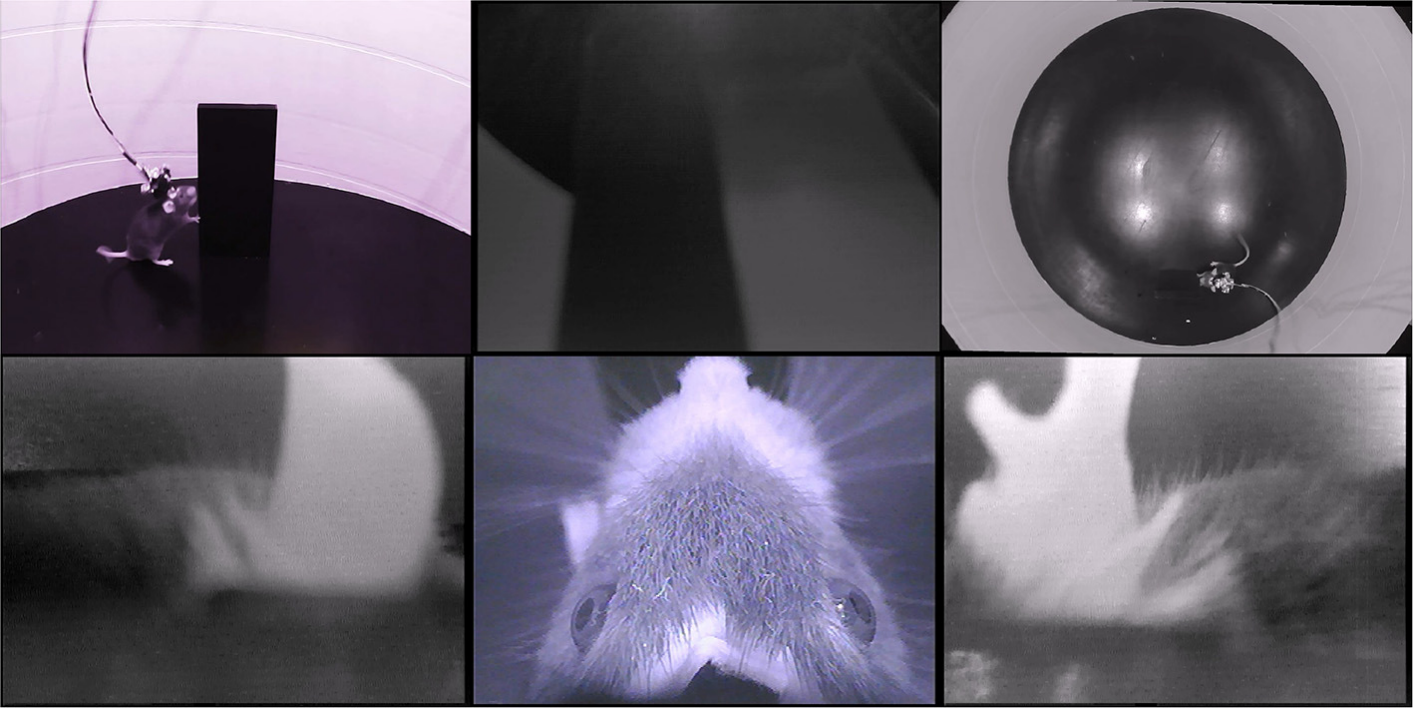
Example data from the four-camera headset. An example frame from Supplementary Video 1, showing the webcam views of a mouse exploring an open arena, from the side (top left) and above (top right), and the video signals from the four-camera headstage-integrated headset: left ear camera (bottom left), right ear camera (bottom right), overhead camera (bottom middle), and forward-facing camera (top middle). Note that the overhead camera video signal is of higher quality than the front-facing or ear-facing videos, due to their filtering.

First, we describe a four-camera headset, designed as an attachment for the Intan 32-channel electrophysiology headstage. The four cameras provide an independent view of each of the two ears, an overhead view of the eyes, whiskers, and rhinarium, and a forward-facing view of the visual scene. Figure 1 and Supplementary Video 1 show an example of the signals from this four-camera headset as a mouse explores an object in an open-field arena. Pupil diameter, gaze direction, whisking, and rhinarial movements can be measured from the overhead camera, and ear movements can be measured from the ear-facing cameras.

**Figure 2:**
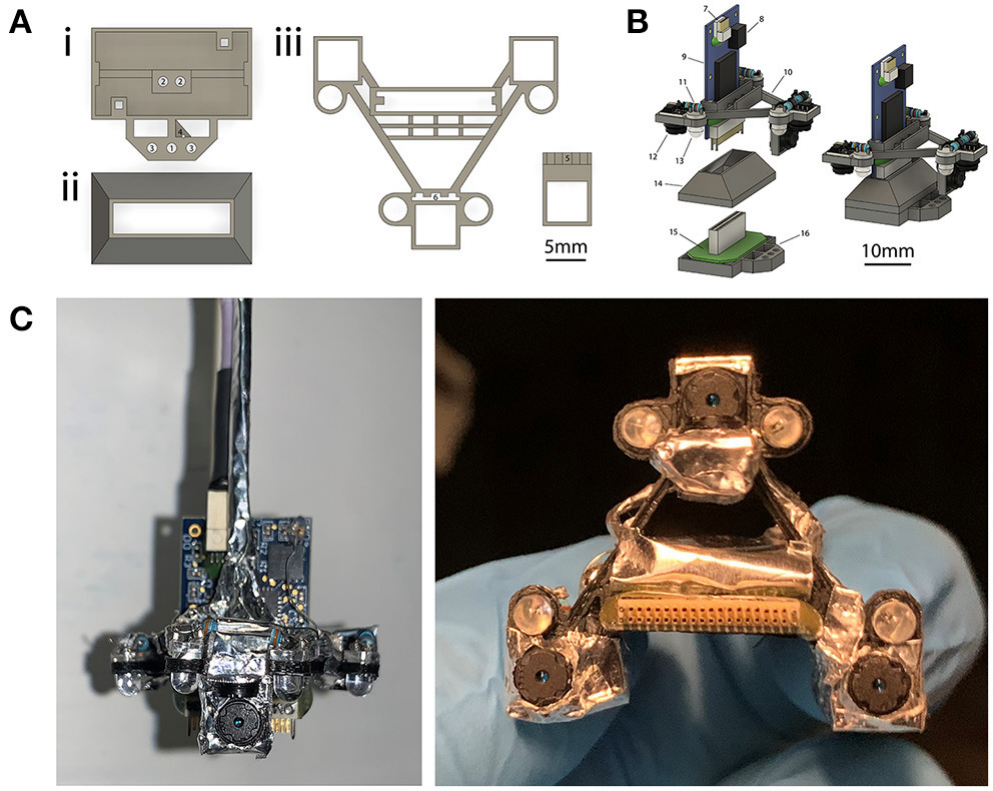
Four-camera headset design. **(A)** 3D-printed components used for implants and headsets. (i) Implant base that is implanted on the head. A Neuralynx EIB is attached to the implant base. (ii) Protective cap that snaps onto the implant to allow for social housing when the camera assembly is not in use. (iii) Camera carrier that holds the cameras and their associated LEDs. The ear-facing cameras are each paired with a single LED for illumination, whereas the overhead camera has two LEDs (for illuminating the left and right side of the mouse's face), and the forward-facing camera does not have an LED. The forward facing camera holder is attached to the main camera carrier component after printing. Numbered labels indicate (1) tetrode drive screw, (2) tetrode guide rails, (3) optional independent camera mount, (4) tetrode guide cannulae, (5) forward camera holder (6) receiving slot for forward camera holder. For further details on labels 1–4 see section Methods: Implants, for labels 5–6 see section Methods: Four-Camera Headset, Construction. **(B)** Rendered view of the components in **(A)**, separately at left and assembled at right. Numbered labels indicate (7) SPI cable connector, (8) integrated accelerometer, (9) Intan 32-channel headstage, (10) camera carrier, (11) resistor, (12) camera module, (13) IR LED, (14) protective social cap, (15) Neuralynx EIB, and (16) implant base. **(C)** A view of the headset assembled around the headstage from the front (left) and underneath (right).

The four-camera headset weighs a total of 1.64 g (Table 1). When combined with an electrophysiology headstage, and an implanted tetrode microdrive, electrode interface board (EIB), and an implant base (Figure 2Ai), the total weight of the system is 3.51 g. Camera headsets consisted of analog camera modules assembled on a 3D-printed camera carrier (Figure 2Aiii), which attaches to the Intan headstage (Figures 2B,C). Integrating cameras with the headstage in this way allows electrophysiology, accelerometer, and video signals to be collected with a single mechanical attachment to the head (at the EIB), which simplifies experimental setup and reduces the overall weight. This design also allows for stereotactic precision and reproducibility of camera views across animals and implants by combining the fixed dimensions of the implant with the stereotactic targeting of the electrodes. Additionally, the focal plane of each camera can be easily adjusted to accommodate for surgical or anatomical jitter encountered between animals (see section “Reproducibility and Precision” below for more details). When the factory-installed lens on the camera modules is maximally extended, the field of view is ~12 × 9 mm at the level of the focal plane, which is ~12 mm away. By screwing the lens all the way in, the camera's focal length increases toward infinity, with a corresponding increase in field of view.

**Table 1:** Weight comparisons.

**Number of cameras**	**1**	**2**	**4**	**8**
RS-306 camera alone (g)	0.18	0.36	0.72	1.44
RS-306 camera assembly (g)	—	0.68	1.64	2.16
RS-306 Electrophysiology/camera assembly (g)	—	2.56	3.51	4.03
Adafruit 1937 camera alone (g)	0.5	1.0	2.0	4.0
Adafruit camera assembly (g)	1.28	2.55	5.10	10.24
Adafruit Electrophysiology/camera assembly (g)	4.28	5.55	8.10	13.24

*Camera assemblies include the cameras, structural carrier, LEDs, resistors, wiring, and connectors. Adafruit assembly also includes IR mirrors. Our 2-camera and 4-camera assemblies correspond to the configurations used in Figures 4C, 2A, respectively. We have not constructed an 8-camera headset but rather estimated weights from the parts shown in Figure 6. “—” indicates not tested, since we only constructed multi-camera headsets. Electrophysiology/camera assembly includes a camera assembly, electrophysiology headstage (Part #17), EIB (Part #32), and implant base (Part #27). Weights for the Adafruit camera assembly are from the single-camera system described in Meyer et al. (2018), which we extrapolated for multiple cameras. The weight of the Adafruit Electrophysiology/camera assembly was estimated by adding an electrophysiology headstage (Part #17, 1 g) and flexDrive (Voigts et al., 2013, 2 g), but does not include the weight of a custom aluminum implant base (Meyer et al., 2018). Mass of cement and/or skull screws is not included. An adult mouse can support ~4 g at most (Voigts et al., 2013)*.

Each camera requires three electrical connections: a power, ground, and signal. With this headset design, the four cameras share power and ground connections, thereby only requiring a six-conductor tether (or six individual wires). The common ground introduces interference in the video signals caused by high-frequency crosstalk, but this interference can be removed through the use of simple low-pass filters (see section “Powering and Signal Conditioning” below for more details). One camera can remain unfiltered, yielding a higher quality video signal. We chose the overhead camera in this case, to achieve finer detail of the eyes, whiskers, and rhinarium.

This headset design has some drawbacks compared to the two-camera headset described below. Once assembled, the camera positioning is not adjustable, requiring iterations of building, and testing to optimize camera angles for each use-case. Additionally, while this headset arrangement allows for a large view of multimodal behavior, the overhead perspective may miss a portion of the pupil in rare cases of extreme ventral gaze. Lastly, because the headset is fully integrated, if one component were to fail, it may be difficult to individually replace it without rebuilding the entire headset (although the camera modules can be reused).

### Adjustable Two-Camera Headset

**Figure 3:**
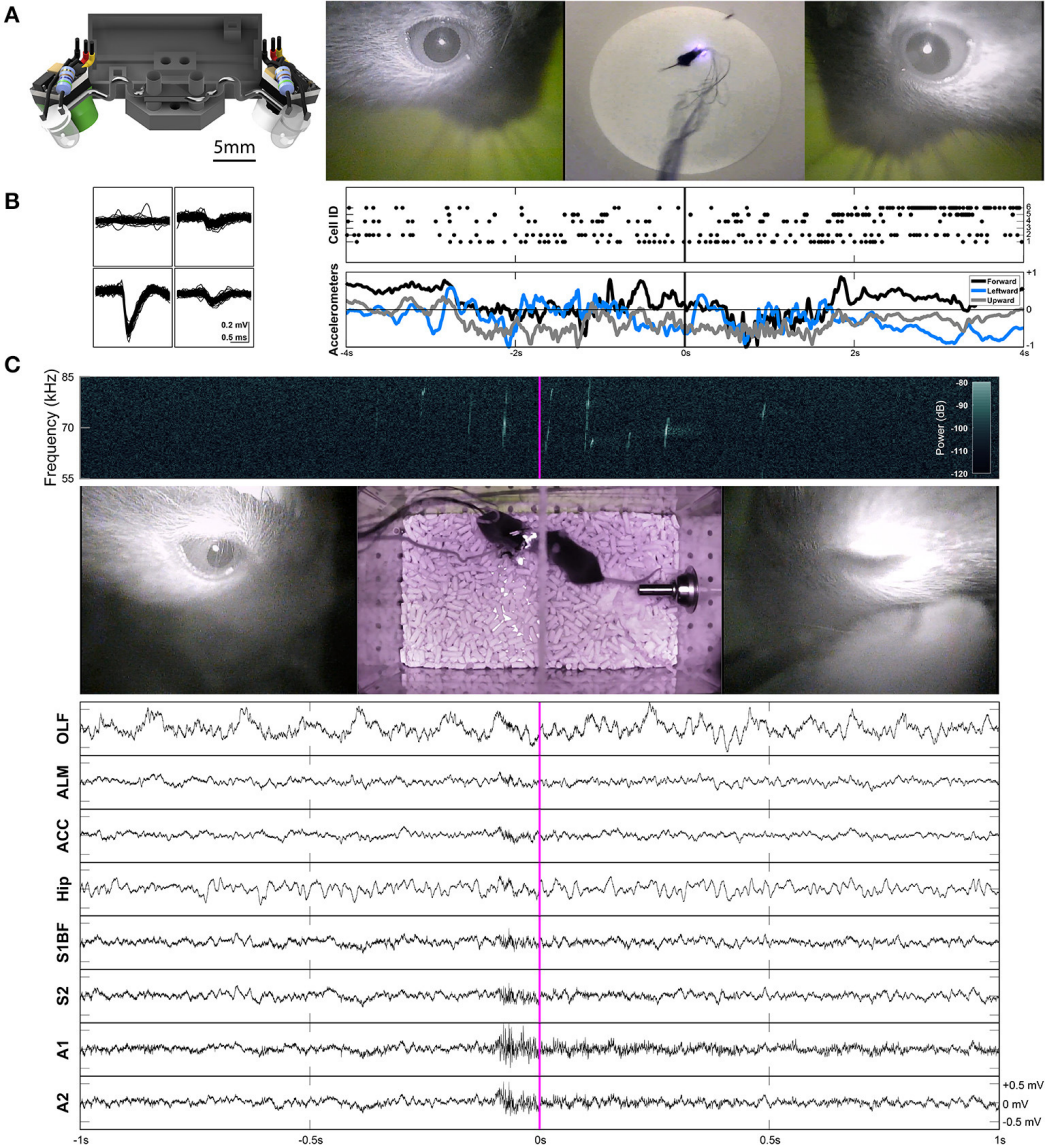
Two-camera headset, and two example applications of freely-moving behavior and electrophysiology. **(A)** Rendered view of the two-camera headset (left), and an example frame from Supplementary Video 2 showing the signals produced by this headset (right). A collimating lens (revealed on the right side of the render) was used for some experiments, but is not necessary for capturing all eye movements. **(B)** Example waveforms across the four channels of a tetrode shown for an individual unit (cell ID 1) recorded in anterior lateral motor cortex (ALM) during prey capture behavior. Rasters of cells firing and the accelerometer channels are plotted together (right) for the corresponding frames shown in **(A)**. The accelerometers are built into the Intan headstage. **(C)** An example frame from Supplementary Video 3. The left eye is closed (due to a blink). A spectrogram (top) shows ultrasonic vocalizations occuring during courtship behavior. Continuous LFP traces from various recording locations are shown below. OLF, olfactory bulb; ALM, anterior lateral motor cortex; ACC, anterior cingulate cortex; Hip, CA1 of the hippocampus; S1BF, barrel field of primary somatosensory cortex; S2, secondary somatosensory cortex; A1, primary auditory cortex; A2, secondary auditory cortex.

Next, we describe an adjustable two-camera headset as shown in Figure 3A, designed to consistently view the entirety of both pupils, and to capture all eye movements in higher detail. To provide the highest-quality video signal, both cameras have independent signal, power, and ground connections for full signal integrity (see section “Powering and Signal Conditioning” for more details).

While this headset is not necessarily integrated with an electrophysiology headstage, it can be easily paired with one. Example waveforms and rasters of unit activity recorded in anterior lateral motor cortex (ALM) during prey capture behavior are shown in Figure 3B and Supplementary Video 2. Single neuron recordings were stable across many days. Raw continuous traces of local field potentials (LFPs) recorded from several different brain areas during courtship behavior are also shown in Figure 3C and Supplementary Video 3 (see sections “Implants,” “Surgery,” and “Experimental Conditions” for more details).

**Figure 4:**
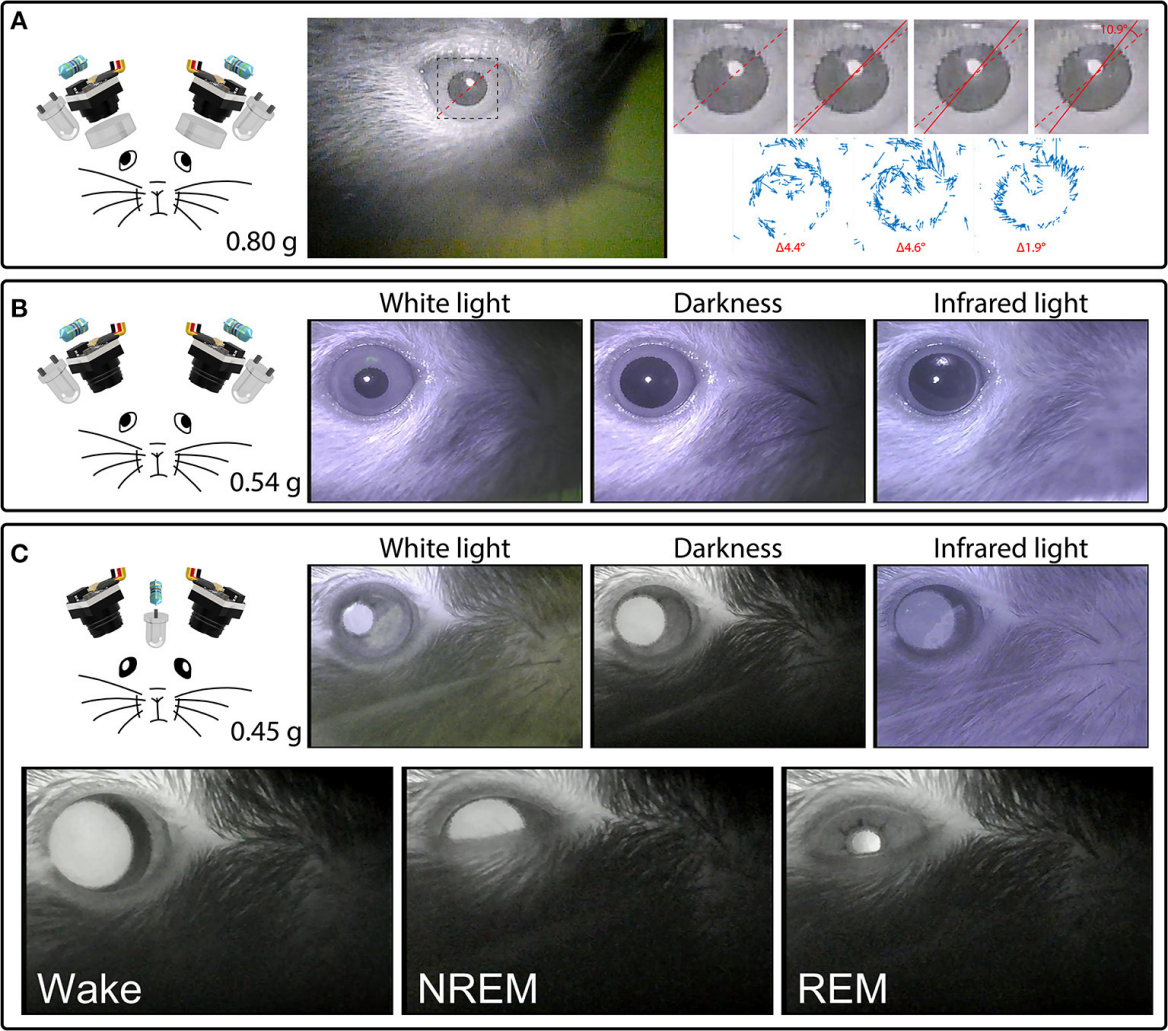
Headset components pictured with their total weight (left) and examples of their video signals (right). **(A)** A camera headset consisting of two camera modules, resistors, external IR LEDs, and collimating lenses (left), and an example frame from Supplementary Video 4, showing the right eye (middle). The inset marks the cropped location of the enlarged images of the pupil (right) from four consecutive deinterlaced frames of Supplementary Video 4 at 60 Hz, with optic flow vector fields between successive frames shown underneath. Dashed red lines mark the orientation of prominent serrations across the pupillary rim of the iris in the first frame. Solid red lines track these serrations in consecutive frames. See Supplementary Video 4 for the full video. Optic flow was calculated in Matlab using the Lucas-Kanade derivative of Gaussian method with a noise threshold of 0.0005, and plotted with a scale factor of 100. **(B)** A camera headset consisting of two camera modules, resistors and external IR LEDs (left), and example frames of the video signals produced under white houselights, no houselights (darkness), and infrared houselights (right). See Supplementary Video 4 for example footage under white houselights. **(C)** A camera headset consisting of two camera modules, one resistor, and one IR LED chronically implanted above a cranial window for infrared back-illumination pupillometry (iBip), and example frames of the video signals produced under white houselights, no houselights (darkness), and infrared houselights (top right). See Supplementary Video 4 for example footage under white houselights, and Supplementary Video 5 for example footage in darkness, displaying the fundus, and optic disc. Example frames of wake, NREM sleep, and REM sleep recorded in the nest of the home cage (bottom). See Supplementary Video 6 for the full video.

With the camera module's factory-installed lens fully extended and focused on the surface of the iris, these cameras can detect cyclotorsion of the mouse eye. Examples of this behavior can be seen in Figure 4 and Supplementary Video 4, which show torsional rotations of up to 11° in extent, at rotational rates of up to 4.6°/s. During periods of active movement, these rotational events occurred frequently, on average at 0.3 events per second in this example mouse.

We tracked cyclotorsion using the unique pattern of natural serrations of the mouse eye in a manner similar to iris-registration software used to track torsional movements in humans during laser eye surgery (Shen et al., 2010). These serrations appear above the lens, at the pupillary rim of the iris, and vary between individual mice. Tracking these serrations allows torsional position to be determined without the use of more invasive techniques, such as limbal markings (Shen et al., 2010), scleral search coils (Robinson, 1963), or other magnetic implants (Schwarz et al., 2013). Our initial design used a collimating lens as shown in Figures 3A, 4A, which was subsequently adopted by others (Michaiel et al., 2020). We have since found that this lens is unnecessary for capturing all eye movements, and is not included in the final design (Figure 4B).

While epi-illumination of the eyes with external IR LEDs provides a consistent video signal across lighting conditions as shown in Figure 4B, infrared back-illumination pupillometry [infrared back-illuminated pupillometry (iBip); (Yüzgeç et al., 2018)] is an alternative approach that can be used with our system. In this configuration, an IR LED is implanted over the skull and back-illuminates the pupil from inside the head. This allows the monitoring of the pupil and torsional position using fewer LEDs and resistors, as shown in Figure 4C and Supplementary Video 4 (see sections “Implants” and “Surgery,” for more details). This method of illumination reveals structures that are not otherwise visible through epi-illumination: prominent natural markings of the iris as shown in Supplementary Video 4, which can provide large and obvious markers for tracking torsional position, and the fundus, retinal vasculature, and optic disc as shown in Supplementary Video 5, which provide the necessary anatomical landmarks to translate the absolute position of the iris into true retinal coordinates. Additionally, utilizing iBip with this system is well-suited for providing robust pupillometric monitoring across both wake and sleep in natural freely-moving conditions. Figure 4C and Supplementary Video 6 show examples of wake, NREM sleep, and REM sleep—characterized by their behavioral hallmarks—from an unrestrained mouse in the nest of the homecage (Yüzgeç et al., 2018; Blumberg et al., 2020).

Lastly, the mounting system of this headset uses steel wire and cement, rather than a 3D-printed carrier, which allows the camera angles and positioning to be adjusted by bending the steel wires. Supplementary Video 7 shows an example of an alternative camera perspective generated by simply bending the wires (for a more dorsal view of the eye and whiskers), illustrating how this mounting system is useful for testing different camera angles in pilot experiments (in this case, as a mouse performs a jump). While this mounting strategy of bending wires lacks exact reproducibility across headsets, reproducibility of camera views across animals and implants can still be achieved for an individual headset by combining the fixed dimensions of the implant with the stereotactic targeting of the electrodes and *post-hoc* calibration of their geometric positioning. Ultimately, once the spatial arrangements of the components have been optimized for your desired signal(s), the headset could be adapted to use a 3D printed carrier, similar to the four-camera headset described above. Even during rapid behaviors such as jumping, we did not observe any motion artifacts in recorded video (e.g., Supplementary Video 7), although the steel wire headset is susceptible to minor motion artifacts in the case of a high-speed collision with a wall (as in Supplementary Video 2). We did not observe motion artifacts with 3D-printed carriers, nor did the camera headsets introduce any motion artifacts into electrophysiology signals.

For either headset configuration, the headstage can provide power and ground connections of the correct voltage for the cameras, reducing the tether requirements even further, but at the cost of introducing appreciable levels of 60 Hz noise into the electrophysiology channels. The level of noise with headstage-provided power is unacceptable for LFP recordings, and for small-amplitude single neuron recordings, but could allow isolation of large-amplitude single neurons. We therefore recommend powering the cameras through the tether from an external source to ensure high-quality and noise-free electrophysiology.

## Discussion

All animals move. Whether these are active sensing behaviors such as saccades or whisking, or large-scale movements directed at broader goals such as capturing prey or returning home, the effects on sensory input are profound. Movement also has a substantial impact on brain activity even beyond the directly-involved sensory and motor areas (Musall et al., 2018; Stringer et al., 2019; Salkoff et al., 2020). To understand how the brain underlies both sensation and action during freely-moving behavior, whether natural behavior or choice tasks, will therefore require precise measurement of the movements of the eyes, ears, whiskers, and rhinarium.

Here we have presented a head-mounted multi-camera system for the simultaneous recording of multiple body parts, sensory structures, and the visual scene, along with high-density electrophysiology in freely-moving mice. The core components are lightweight (180 mg) and inexpensive (US$23) analog camera modules with a number of advantages that increase the flexibility of implementation compared to previously published alternatives. Their size, weight, and cabling advantages allow multi-camera headsets to monitor more sensory structures and capture more of the visual field, while their low impact on behavior enables chronic monitoring under natural conditions across the sleep-wake cycle. Additionally, the system can provide high-precision tracking of eye movements, such as cyclotorsion, and allows the absolute position of the eyes to be translated into true retinal coordinates via visualization of the fundus and optic disc. Together, these features allow the alignment of neuronal activity with both motor events and retinotopically mapped visual stimuli in freely-moving animals.

Although we provide examples of specific configurations for monitoring certain body parts and views (i.e., the eyes, ears, whiskers, and binocular visual field etc.), we anticipate that this system will be adapted to best suit specific experimental goals using the strategies and considerations presented below.

### Size, Weight, and Multi-Camera Headsets

**Figure 5:**
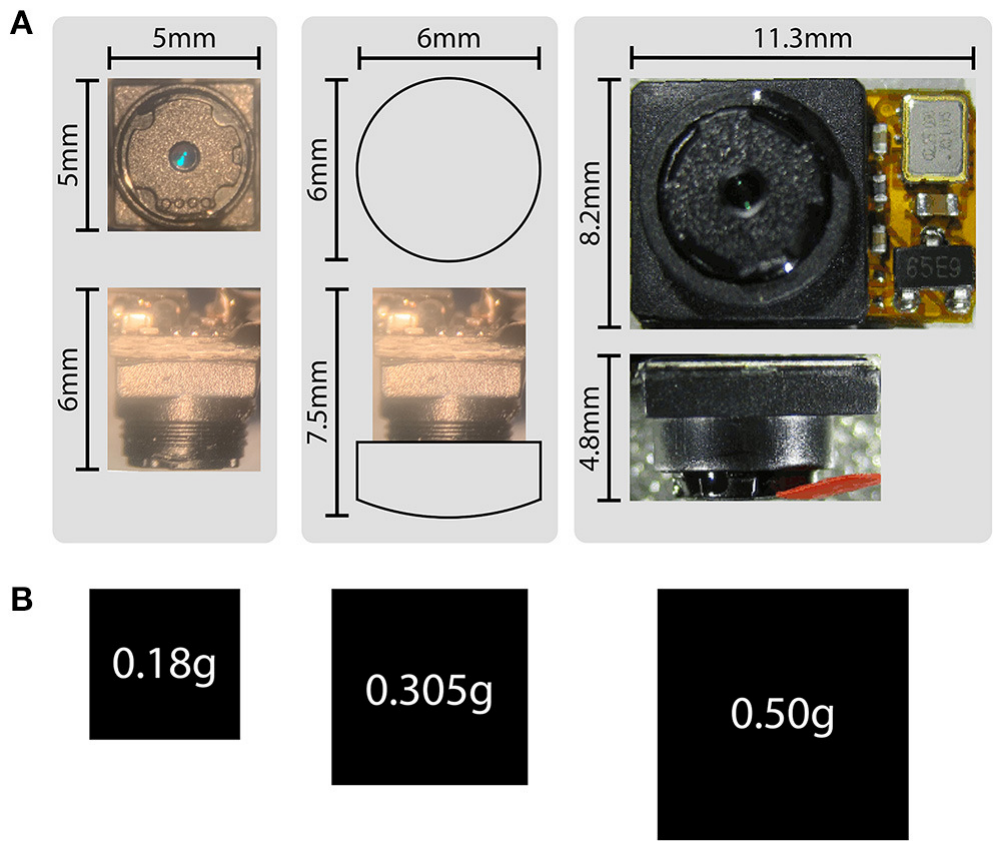
Size and weight comparisons. **(A)** Size comparisons between the analog camera modules (left), analog camera modules with an additional collimating lens (middle), and an Adafruit 1937 picam camera module (right). The factory-installed adjustable lens has 1 mm of travel, and is shown maximally extended (left, for a total camera depth of 6 mm), and minimally extended (middle, for a camera depth of 5 mm, or 7.5 mm with the additional collimating lens). Broader views of the camera modules are also shown in Appendices 1, 2. **(B)** Weight comparisons represented by area for the corresponding camera modules shown in **(A)**.

Size and weight are perhaps the most crucial aspects of head-mounted devices, and together are significant advantages of this system compared to previously published alternatives. Because analog camera modules forgo on-chip digitization of the video signal, ADC hardware components are eliminated from the design at the level of the animal, resulting in a significant reduction of both size and weight. At 5 × 5 × 5 mm and 180 mg, the size and weight of these camera modules serve to minimize their effects on mouse behavior. Figure 5 shows the differences in size and weight between these camera modules (the LeeChatWin RS-306) and the Adafruit 1937 camera, a leading alternative for behavioral monitoring of freely-moving mice (Meyer et al., 2018).

**Figure 6:**
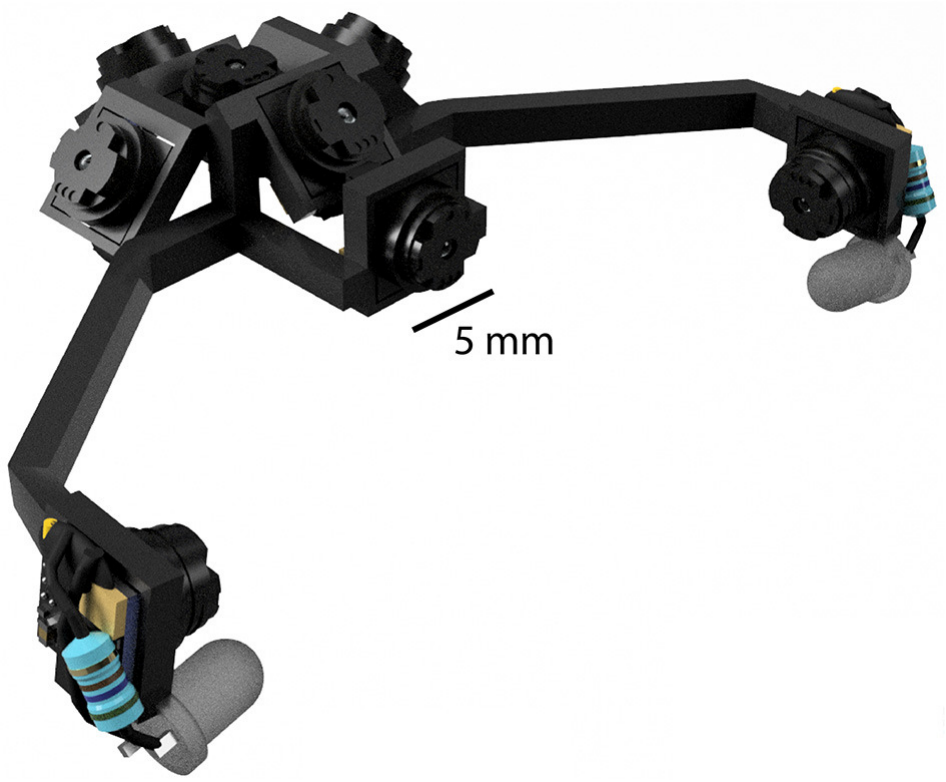
An example rendering of an 8-camera headset designed to capture the binocular visual field while monitoring the eyes. Although a mouse could additionally support the weight of an electrophysiology headstage and EIB, the headstage would necessarily occlude some portion of the visual field.

The primary benefit of this is to reduce the load carried by the animal, which in turn leads to a number of corollary benefits. First, cameras can be much more readily incorporated with larger and heavier hardware, such as electrophysiology headstages, and positioned in much closer quarters due to their size and flexible cabling options. Second, more cameras can be used to monitor additional body parts and classes of movements. Using more cameras is likely to be important, as recently described brainwide correlates of high-dimensional motor variables show the importance of capturing as much behavioral information as possible (Musall et al., 2019; Stringer et al., 2019). Third, more cameras can be used to capture more of the visual field, and at the same time can be packed closer together to obstruct less of the visual field. Fourth, mirrors are not required to achieve desired viewing angles, because the cameras themselves are light enough to extend distally from the head. This greatly reduces the complexity of headset designs as well as reducing overall weight. All together, these advantages allow the construction of multi-camera headsets to be much more feasible, as illustrated in Figure 6 which shows an example rendering of an 8-camera headset designed to capture the entirety of the binocular visual field while also monitoring both eyes.

How many cameras could a mouse support? There is no fixed maximum load for head-mounted devices, because capacity depends on the size and strength of each individual. Moreover, the impact to mouse behavior increases smoothly with load rather than suddenly reaching a threshold, and the negative consequences will depend on the behavioral task in each experiment. Nevertheless, it is straightforward to compare the maximal number of cameras across systems for a given load limit. For example, a load limit of 4 g would permit 8 cameras and a 32-channel electrophysiology headstage (Table 1), whereas with the Adafruit 1937 only 1 camera and a headstage would be possible (Meyer et al., 2018).

The four-camera electrophysiology headset described above, including the electrophysiology headstage, weighs a total of 3.5 g, for minimal effects on natural behaviors such as prey capture and vocal interaction. While the RS-306 camera is significantly smaller and lighter, the Adafruit 1937 camera has superior temporal resolution, capable of capturing digital 640 × 480p frames at 90 Hz. The Adafruit 1937 can also be configured to capture 1,080p frames at 30 Hz, or 720p frames at 60 Hz. Taken together, the size and weight advantages of our system are significant, but the lower temporal resolution of the analog cameras (60 Hz compared to 90 Hz with the Adafruit 1937) presents a trade-off that depends on individual experimental requirements.

### Cyclotorsion

Natural cyclotorsion of the eye has been observed in pigeons (Benjamins and Huizinga, 1929), chickens (Schwarz et al., 2013), rats (Preyer, 1882; Welker, 1964; Carvell et al., 1991; Wallace et al., 2013), and primates (Wells, 1794; Helmholtz, 1925), but to our knowledge has not previously been described in mice. Cyclotorsion is known to interact with the vestibular system to provide gaze stabilization as the head rotates about an axis of fixation or an object of smooth pursuit (Tweed and Vilis, 1987; Crawford et al., 1991; Angelaki and Dickman, 2003). The occurrence of cyclotorsion events up to 11° in mice highlights the importance of capturing all eye movements for studies of vision in freely moving animals (Ballard, 1991), and its necessity for studies that seek to relate retinotopic receptive fields to visual stimuli or behavior (Cooper and Pettigrew, 1979).

### Absolute Retinal Position and Retinotopic Mapping of Visuotopic Space

The absolute position of the iris corresponds to the absolute position of the retina (Felius et al., 2009). However, the features of the iris are unique between individuals and lack any predefined location around the pupillary rim. Translating the torsional orientation of the iris into the torsional orientation of the retina therefore requires a geometric calibration between at least two points on both the iris and the retina to establish their alignment. This requires anatomical landmarks on the mouse retina to establish its torsional orientation in reference to the camera's position. With the lack of a visible macula in mice (Volland et al., 2015), the optic disc and retinal vasculature may be the only readily visible anatomical landmarks on the retina with which torsional position can be established (Parsa and Kumar, 2013). Using our system with iBip reveals the optic disc and retinal vasculature, and allows them to be visualized together with the features of the iris (Supplementary Video 5). This provides the necessary anatomical landmarks from the common perspective of the camera, such that the torsional position of the iris can be used to determine the torsional position of the retina. Calibration of anatomical retinal coordinates to absolute visuotopic coordinates requires an additional step to anatomically identify the visual midline (as described in detail by Sterratt et al., 2013). With the use of modern inertial measurement devices and video tracking to determine the camera's absolute position and orientation, this calibration can then be used with the absolute position of the pupil to project retinotopic coordinates onto visuotopic space (Sterratt et al., 2013; Wallace et al., 2013; Matthis et al., 2018). Utilizing iBip with this system is therefore particularly well-suited for experiments seeking to link neuronal activity in retinotopically organized brain areas with visual stimuli in freely moving conditions.

### Tethers

For head-mounted video, tethers will likely remain necessary until further advances in power and high-bandwidth telemetry or local data storage become available. As a mouse turns, tethers accumulate torque which strains their free movement and may bias their decisions. Importantly, since this system uses individual wires for signal and power connections, there are many available options of fine flexible wire to choose from as opposed to the flat ribbon cables of the Adafruit 1937 camera. Additionally, they can be readily integrated into slip ring commutators and pulleys to relieve strain from the tethers, which may prove more difficult with a ribbon cable. Using modern commutators which utilize close-loop systems to provide active accommodation could also be implemented to relieve any strain of the electrophysiology and camera tethers, as well as optical fibers (Hoshino et al., 2020). Active counterbalancing of the tethers through closed-loop tracking and motorized pulleys should also be possible, however we have not yet tested motorized commutators or pulleys with this system. Lastly, these tethers can directly power LEDs, which allows them to be easily integrated into headsets without requiring additional tethers or electrical connections to additional implanted hardware.

### Reproducibility and Precision

The precise positioning of electrodes and cameras is limited by the surgical jitter of implants and individual anatomical differences across mice. The solution we described here is to integrate conventional stereotactic targeting with 3D-printed implant design. A promising framework for these types of implants is the RatHat, which is an open-source self-targeting 3D-printed brain implant system (Allen et al., 2020). Manual adjustment of the fine focus of the individual camera modules can also help compensate for remaining differences in focus encountered between animals. Taking note of the markings on the face of the lens when adjusting between animals is helpful for reproducibility in this case.

### General Design Guidelines

Other undesirable consequences of all head-mounted devices include partial occlusion of the visual field, interference with sound localization due to sound shadows, and interference with access to apparatus components such as nosepokes. For these reasons, camera headsets should be designed with specific experiments in mind, to balance the benefits of the desired behavioral and physiological signals with their impact on sensation and behavior.

When customizing the camera set-up to target different camera views, electrode locations, or other hardware, we recommend the following design considerations. First, determine the locations of optical fibers or microinjection ports, which require open access during experiments. The EIB should then be placed where it doesn't impede this access. A different choice of EIB or the use of silicone probes will likely affect this placement. The implant base should then be designed and positioned to accommodate the EIB and other hardware. Finally, design the camera carrier based on how it will mount to the implant base or EIB, in order to target the cameras at the desired viewpoints.

### Future Directions

We envision a number of extensions for this system. Firstly, utilizing open-source frameworks for 3D-printed stereotaxic implants, such as the RatHat (Allen et al., 2020), will allow the targeting of electrodes, cameras, optical fibers, and other components to be unified within and across animals, implants, experiments, and labs. The use of high-density silicon probes, such as neuropixel arrays (Steinmetz et al., 2020), will dramatically increase the number of neurons that can be recorded alongside head-mounted video, and a common framework for 3D stereotaxic implants will therefore enable more replicable designs, experiments, and analyses, as hardware components continue to evolve and improve.

Integrating such a framework into open-source libraries for experimental design, such as Autopilot (Saunders and Wehr, 2019), will also allow for increased ease of use and replicability of experimental setups and analysis. These efforts to standardize data organization may also serve to streamline experiments which require several processes of analysis and reconstruction techniques using various software packages, such as Kilosort (Pachitariu et al., 2016), DeepLabCut (Mathis et al., 2018), or Retistruct (Sterratt et al., 2013).

Finally, we have not explored the upper limits on the number of camera modules that can fit on the head and be supported by a mouse without noticeably impacting behavior. Improvements in implant design and tether choice will improve efficiency and will likely continue to increase the number of devices that can be included. Optical imaging of neural activity using these sensors may also be a promising approach. A widefield miniscope using this analog camera module could be integrated with behavioral camera arrays, like those in Figure 1 for example, to investigate large-scale brain activity in freely moving conditions.

## Methods

### Preparation of Camera Modules

We used LeeChatWin RS-306 miniature analog camera modules (Part #1), but other miniature analog camera modules with similar specifications are available from a range of manufacturers. Before modifying the cameras in any way, we ensured that they produced a clean and stable video signal out of the box by powering the camera's attached barrel cable with a 12V DC power supply and connecting its RCA composite video connector to a display. We then removed the infrared filter as described in Appendix 1, cleared away excess rubber as described in Appendix 2, and stripped insulation from the leads as described in Appendix 3.

### Four-Camera Adjustable-Focus Headset

#### Construction

All 3D-printed components (Figure 2A) were designed and rendered with Autodesk Fusion 360 software (Part #2), prepared using Cura (Part #3), and printed with polylactic acid (PLA) on a Monoprice Maker Select Plus 3D Printer (Part #4).

We 3D-printed a camera carrier (Part #5) as shown in Figure 2Aiii. The carrier was inspected and tested to ensure the camera modules would properly fit into each camera holder as described in Appendix 7. We then applied a thin coat of superglue to the grooves of the forward camera holder (marked 5 in Figure 2Aiii), and inserted it into the corresponding slot in the camera carrier (marked 6 in Figure 2Aiii, also see photo in Appendix 7).

To create the headset, we first prepared a wiring harness with measured segments of wire needed to power the IR LEDs and camera modules on the headset (Appendix 4). We then soldered the prepared wires to each camera module (Appendix 5), shielded them with aluminum tape (Appendix 6), and cemented them onto the headset (Appendix 7). We then inserted the IR LEDs into the receiver holes in the carrier and soldered the leads to their respective camera module (Appendix 8). The IR LEDs provide constant and sufficient illumination of the eyes, ears, or face during experiments regardless of head position, while this illumination remains invisible to the mice. Next, we soldered the power wires to a single power tether, and the ground wires to a single ground tether (Appendix 9). We then applied the last piece of shielding (Appendix 10) and attached the headset to an Intan headstage by inserting the headstage into the receiver slot in the carrier, and then sliding the carrier all the way down onto the headstage (Appendix 11). A friction fit is sufficient to keep the headset firmly mounted to the headstage indefinitely. Repeated attaching and detaching of the headset to headstages could cause wear and tear and is not recommended. Finally, we electrically connected all the shields and pinned them to the local ground of the headstage (Appendix 12).

#### Powering and Signal Conditioning

We terminated the ends of the signal, power, and ground tethers with male jumpers for easy connection/disconnection. We used a +3.3V terminal on an RHD2000 USB interface board to power the cameras for use with or without accompanying electrophysiology, but any +3.3V power supply should work. The cameras could optionally be powered directly by the headstage as well, reducing the number of wires in the tether and still allowing accelerometer signals to be acquired, but at the cost of introducing noise on the electrophysiology channels.

**Figure 7:**
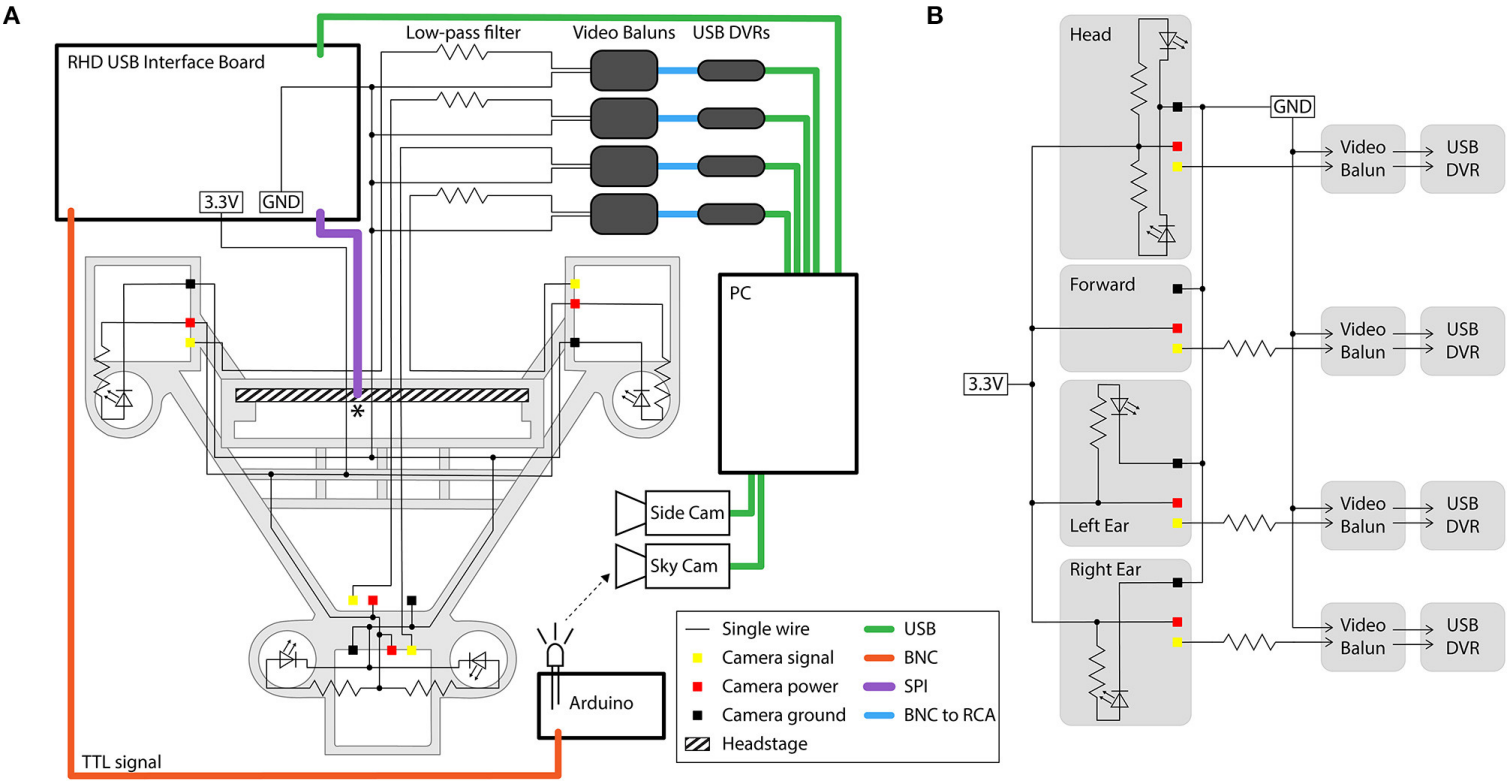
Circuit diagram and connections for the four-camera headstage-integrated headset. **(A)** The camera carrier (same as Figure 2Aiii) is shown in the background in gray. The RHD 32-channel headstage is represented by the hatched rectangle, and is attached to its associated SPI cable represented by the purple line at the point marked *. TTL pulses sent to the RHD 2000 USB Interface Board are relayed through a BNC cable (orange) to an Arduino Uno for synchronization of electrophysiology and video signals by illumination of an IR LED in view of the webcam placed above the arena (the SkyCam). Yellow, red, and black squares represent the soldering locations on the camera modules for the signal, power, and ground wires. Low-pass filters are constructed on a breadboard, and are then connected to video baluns through a BNC connection. From the video baluns, the signal passes through a BNC to RCA connection (blue) to the USB DVRs. Green lines represent USB cables. Black dots indicate logical connections of signals and components; for true solder locations, see the instructions in the Appendices. **(B)** Conventional circuit diagram for powering and signal processing of the analog cameras, same circuit as shown in **(A)** but without illustration of cable type or spatial layout.

The 4 cameras can share power and ground connections, thereby using only 6 wires, but the common ground introduces interference caused by high-frequency crosstalk (producing chrominance and dot-crawl noise). If additional wiring is not a consideration, this interference can be avoided by using independent power and ground connections for each camera (i.e., 12 wires for 4 cameras). Alternatively, the interference can be removed with simple low-pass filters (we used a 560 Ω resistor in series with each video signal). We constructed these filters on a separate breadboard for the camera signals, and integrated it into the system as shown in Figure 7. One camera signal can remain unfiltered, yielding a higher quality video signal.

The filtering eliminates the crosstalk between the cameras produced by the common ground loop, but comes at the cost of losing the chrominance signal and the high frequency portion of the luminance signal. Therefore, color information is lost and a reduction in the luminance of the signal causes a dimming of the video. The drop in luminance can be compensated for by increasing the intensity of LED illumination (by changing the value of the current-limiting resistor for each LED to produce sufficient brightness). We found that filters using a 560 Ω resistor effectively removed crosstalk without too much dimming of the video signal, but this filtering could be affected by stray capacitance in the system and the optimal resistance value may therefore differ across implementations.

Finally, we pass the filtered signal and ground for each camera through individual video baluns (video signal isolators, Part #6) before being connected to USB video capture cards (DVRs) for digitization of the video signal. The video baluns were used to isolate a second ground loop that would otherwise normally occur at this point in this system, produced by having two paths to ground: one to the ground of the camera power supply and one to the ground of the computer.

### Adjustable Two-Camera Headset

#### Construction

We first applied a small amount of super glue (Part #7) for strain relief between the wires at the base of the prepared camera modules so they were secured for long-term use. We then soldered an independent signal, power, and ground tether (Part #8) to each camera module. A 560 Ω resistor (Part #9) was soldered at this location to the power wire, as well as an additional wire to the ground tether, for powering an illumination LED (see below). Shrink tubing was then applied to these three junctions (Part #10). For some experiments, a 6 mm collimating lens (Part #11) was placed onto the front of each camera module using tape (Part #12), but this is unnecessary and not recommended due to the additional 125 mg weight imposed. We then wrapped the camera module with a layer of micropore surgical tape (Part #13) and applied a thin coat of super glue across the surface of the tape.

For adjustable headsets that mounted independently of the headstage, we prepared two 15G hypodermic tubes to a length of 5 mm, and a 20G stainless steel wire (Part #14) to a length of 3 cm. The hypodermic tubes were inserted onto the front two screws of an assembled implant (3 in Figure 2Ai), and the stainless steel wire was cemented between them with super glue, followed by a layer of dental acrylic. For adjustable headsets that mounted with the headstage, we trimmed down a camera carrier (Part#5) using wire cutters, such that only the headstage connector and wire weaving lattice remained. We then placed 20G stainless steel wires through a weaving window on both sides of the carrier and cemented them into place. Once the wires had fully cured in place, we then cemented the two camera modules bilaterally to the stainless steel wires.

For epi-illumination of the eyes, we then cemented 3 mm IR LEDs (Part #15) to the sides of each camera module in a coaxial fashion, and soldered the leads to the power (through the current-limiting resistor) and ground tethers. Supplementary Video 7 shows an example of the video signal that is produced if epi-illumination from an attached IR LED is not utilized. For iBip headsets, we instead prepared wire soldered to the female end of electrical connectors (Part#47), and soldered the free end of this wire to the available free ends of the ground wire and powering resistor of a camera module. The wire was cut to a length to allow the male ends of these connections to be reversibly attached to the connectors of the iBip implants during experiments to power the implanted LED.

#### Powering and Signal Conditioning

The distal ends of the camera signal, power, and ground tethers were then soldered back to their original connections on the factory-provided wiring harness with the power and video connectors. The two cameras were then each powered with an independent 12V DC power supply and connected with RCA cables to USB video capture cards (DVRs, Part #16) for digitization of the video signal. The factory-provided power connector includes a step-down from 12 to 3.3 V.

### Data Acquisition and Processing

Electrophysiology and accelerometer signals were acquired from an attached Intan headstage amplifier (Part #17) with an RHD2000 USB interface board (Part #18) using OpenEphys software (Part #19). The Intan headstage contains 3 integrated accelerometers. We identified single neurons offline using Kilosort spike sorting software (Part #20).

For either headset design, DVRs were connected to a recording PC, and video signals were acquired with Bonsai software (Part #21) at 30 Hz and a resolution of 1,280 × 960. Video from additional webcams (Parts #22, 23) were also simultaneously acquired with Bonsai at 30 Hz and a resolution of 1,280 × 960 or 1,920 × 1,080. We increased the brightness of the ear-facing and overhead camera videos using the color balance function in Bonsai. Video can also be flipped or rotated at this point; for example, we rotated the signal from the forward camera video to compensate for the installation orientation. We also used Bonsai to record timestamps for all captured frames and log them to individual csv files for each camera. To synchronize the video signals with electrophysiology and accelerometer signals, we positioned an IR LED in view of the webcam, and drove it with TTL pulses also recorded by the RHD2000 USB interface board. The LED pulses were detected online during data acquisition with Bonsai in real time. The analog camera modules are not directly triggerable. We note that the DVRs are susceptible to electrical interference, e.g., from nearby power supplies or equipment that can introduce artifacts into the digitized video signal if too close to the DVRs.

The RS-306 camera produces an NTSC analog waveform encoding 4:3 aspect ratio interlaced frames with an optical resolution of 800 × 480 at 30 Hz. We deinterlaced the video offline to remove combing artifacts and recover the full 60 Hz field rate. Analog video is interlaced such that each 30 Hz video frame consists of two 60 Hz fields taken in sequence: the first containing the odd lines of the image, and the second containing the even lines. We used a line-doubling deinterlacing algorithm to separate each frame into two consecutive deinterlaced images consisting of the odd or even field lines, and doubled line width to preserve image dimensions, yielding deinterlaced video at 60 Hz, with an effective single-frame optical resolution around 800 × 240. The horizontal and vertical angles of view are 60° and 45°.

Stereo audio recordings for vocalization experiments were obtained with two Brüel and Kjær 1/4-inch microphones (Part #24) and acquired with a Lynx 22 sound card (Part #25) and Audacity software (Part #26). The transducers of the microphones were positioned at the ends of the cage and angled at 45 degrees to point at the base of the interaction barrier. We delivered white noise bursts through a free-field speaker at the beginning and end of each experiment along with a TTL pulse to an IR LED and the RHD 2000 interface board to synchronize audio and video recordings with electrophysiology and accelerometer signals. For the spectrograms in Figure 3B, we included noise reduction processing in Audacity using a noise profile of 5 s, noise reduction of 19 dB, sensitivity of 24, and frequency smoothing of 12.

### Implants

We tapped the three holes at the front of the implant base (Figure 2Ai, Part #27) with a 00–80 tap, and cut a 00–80 screw (Part #28) to an 8 mm length and screwed it into the center hole (marked 1 in Figure 2Ai) with a washer (Part #29) to serve as a drive screw. We then tapped a 6 mm piece of plastic to serve as a cuff, and mounted it onto the end of the drive screw to create a total height of 1.3 cm from the bottom of the cuff to the top of the drive screw. For iBip implants, an IR LED (Part#15) was cemented to the bottom of the cuff, between the power and ground leads of the LED. The addition of the LED adds 5 mm in height to the implant, and therefore the drive screw and plastic cuff were instead cut to 5 and 4 mm, respectively, to achieve the total height of 1.3 cm.

For tetrode implants, we then inserted two 6 mm 18G hypodermic tubes into the center of the implant base (marked 2 in Figure 2Ai) to serve as guide rails for vertical travel of the base when advancing the electrodes. Two additional 8 mm 00–80 screws were screwed in the remaining holes at the front of the implant base (marked 3 in Figure 2Ai) from the bottom up, to serve as a site for attaching the camera headset if an independent camera attachment site was desired. For iBip implants, electrical connectors (Part#47) were placed in these holes instead, with the female connection site flush with the top surface of the implant, to serve as the connection sites to power the LED during experiments. We then soldered the power lead of the LED to the right connector, and the ground lead to the left connector. Exposed wire and solder were then coated with cement.

For tetrode implants, we inserted two 6 mm 29G hypodermic tubes through the hole in the base (marked 4 in Figure 2Ai), and cemented them in place with dental acrylic. For stainless-steel wire arrays, we inserted 7 mm 19G hypodermic tubes and cemented them in place with dental acrylic near their relative stereotactic locations on the implant base to accommodate two teflon-coated stainless steel wires each (Part #30).

We used small EIB pins (Part #31) to electrically connect individual stainless steel wires to the ground and reference channels on a Neuralynx EIB (Part #32). An EIB (electrode interface board) is a miniature break-out board with a headstage connector (such as an Omnetics connector) and connection points for implanted microwires, to provide a robust interface between a headstage and electrode or tetrode channels. The remaining channels on the EIB were similarly connected to prepared tetrodes (Part #33) or stainless steel wires for tetrode and wire-array implants, respectively. Once connected, we coated these sites with silicone sealant (Part #34). The reference and ground wires were either routed through the drive posts for tetrode implants, or two 19G hypodermic tubes in the case of wire-array implants. The tetrodes or remaining stainless-steel wires were then routed through their respective hypodermic tubes, and the EIB was lowered and flushly screwed into place on the surface of the implant base with two screws (Part #35). Stainless steel wires routed through the same tubes were cut to slightly different lengths so that we could properly identify them during implantation and note their respective channels on the EIB. The implants were then sterilized in 70% EtOH before implantation. Protective caps (Figure 2Aii, Part #36) were attached to implants after surgery.

### Surgery

All procedures were performed in accordance with National Institutes of Health guidelines, as approved by the University of Oregon Institutional Animal Care and Use Committee.

For the experiments described here, we used adult C57bl/6 mice (*n* = 9, both males and females, >3 months of age, weighing 18.5–31 g at the time of surgery) and made craniotomies as described below; these can be customized to the specific needs of different experiments. Mice were anesthetized with isoflurane (1–2%). A craniotomy was created over the right hemisphere, where a skull screw (Part #37) was fastened into place and cemented with dental acrylic. Two additional craniotomies were then created at −1.5 AP, −2 ML, 0 DV, and −2 AP, −2 ML, 0 DV for the ground and reference wires. For implants utilizing infrared back-illumination pupillometry [iBip; (Yüzgeç et al., 2018)], a 2 mm craniotomy was created at 4 AP, 0 ML, 0 DV relative to bregma, and a coverslip was secured overtop using vetbond. For tetrode implants, a small craniotomy was created dorsal to anterior lateral motor cortex (ALM) at 2.5 AP, 1.5 ML, 0 DV (relative to bregma). For wire-array implants, craniotomies were similarly created for recordings in the olfactory bulb at 4.5 AP, 0.8 ML, 0 DV, ALM at 2.5 AP, 1.5 ML, 0 DV, anterior cingulate cortex (ACC) at 1.98 AP, 0.35 ML, −1.8 DV, CA1 of the hippocampus at −2.06 AP, 1.5 ML, −1.5 DV, the barrel field of primary somatosensory cortex (S1BF) at −1 AP, 3.5 ML, 0 DV, secondary somatosensory cortex (S2) at −1.5 AP, 4.5 ML, 0 DV, primary auditory cortex (A1) at −2.9 AP, 4.5 ML, 0 DV, and secondary auditory cortex (A2) at −2.5 AP, 4.2 ML, 0 DV. Sterile saline was immediately applied and maintained at these locations to prevent drying of the dura.

Proper positioning of the cameras, electrodes, and other hardware such as optical fibers or microinjection ports will depend on the body parts and brain areas being targeted. For the experiments described here, EIBs were stereotactically placed with the center of the surface of their base at −5.5 AP, 0 ML, 10 DV from bregma. This was achieved with the fixed dimensions of the implant by positioning the drive screw (or the IR LED in the case of iBip implants) at 4 AP, 0 ML, 0 DV relative to bregma, and ensuring the total height of the implant was 1.3 cm from the bottom of the cuff (or the IR LED in the case of iBip implants) to the top of the drive screw. This positions the EIB centrally over the head and the camera carrier such that the cameras are over the ears and the front of the head.

For tetrode implants, the tetrodes were trimmed at this point so they would pass just beyond the level of the dura when the implant was lowered into its final position. Once at the proper length, we applied a thin coating of antibacterial ointment to the tetrodes, made a small incision in the dura, and slowly lowered the implant and tetrodes into place. We then coated the remaining exposed portion of the tetrodes in vaseline, and cemented the vaseline, cuff, and surrounding surface of the skull with dental acrylic. We then positioned the ground and reference wires just below the dura in their respective craniotomies, and cemented them with dental acrylic along with the drive posts so that no portion of the wires were left exposed.

For wire-array implants, the stainless steel wires were splayed out laterally so the implant could be lowered into its proper position. We then raised the implant 1.5 mm dorsally, and positioned the hippocampal wire vertically just above the dura, and cemented it to the hypodermic tube in this position with dental acrylic. This process was then repeated for the remaining electrodes targeting non-superficial structures. We then made small incisions in the dura at these locations, and slowly lowered the implant into position. We applied dental acrylic to these two sites and the cuff to cement them in place. Once the acrylic sufficiently stabilized the implant, the remaining wires were placed just below the dura in their respective craniotomies, and cemented with dental acrylic so that no portion of the wires were left exposed.

We administered Ketoprofen (5 mg/kg) to reduce post-operative inflammation. The bottom surface of a protective cap (Part #36) was then coated with silicone sealant (Part #34) and snapped over the EIBs of the implant to cure in place. Mice were then given 7 days of post-operative recovery.

### Experimental Conditions

All experiments occurred within an electrically shielded sound-attenuating chamber. Sleep experiments occurred in the nest of the home cage, with no external sources of illumination. Vocalization experiments occurred in the home cage of individually housed male mice. A webcam (Part #22) recorded video directly above the arena (referred to as the “SkyCam”), with illumination from an IR LED light source (Part #38) and 6000K LED Floodlight (Part #39). We placed a perforated clear acrylic barrier (Part #40) between the resident male, who was unimplanted, and an implanted female. Vocalizations were recorded with two overhead Brüel and Kjær 1/4-inch microphones (Part #24).

Prey capture experiments occurred in a 24 inch diameter circular arena with a clean paper floor. SkyCam video of the arena was recorded as described above. Illumination was provided by an IR LED light source (Part #38) and 5,500 K lightbulb (Part #41). An implanted mouse waited in the arena until a cricket was remotely dropped into the arena, and then the mouse chased and captured the cricket (Hoy et al., 2016).

Free field exploration experiments occurred in the same arena as prey capture experiments. We used a webcam (Part #22) to record from one side of the arena, and a dome camera (Part #23) to record from above, with illumination provided by an IR LED light source (Part #42) and 6000K LED Floodlight (Part #39).

## Data Availability Statement

The raw data supporting the conclusions of this article will be made available by the authors, without undue reservation.

## Ethics Statement

The animal study was reviewed and approved by the University of Oregon Institutional Animal Care and Use Committee.

## Author Contributions

NJS developed and tested the system. NJS and MW wrote the manuscript. All authors contributed to the article and approved the submitted version.

## Conflict of Interest

The authors declare that the research was conducted in the absence of any commercial or financial relationships that could be construed as a potential conflict of interest.

## Supplementary Material

Supplementary Video 1 An example video of the four-camera headstage-integrated headset showing the dynamic activity of the rhinarium, whiskers, eyes, and ears of a freely moving mouse. Panels show the webcam views of a mouse from the side (top left) and above (top right), and the video signals from the four-camera headstage-integrated headset: left ear camera (bottom left), right ear camera (bottom right), overhead camera (bottom middle), and forward-facing camera (top middle). Note that the overhead camera video signal is of higher quality than the front-facing or ear-facing videos, due to their filtering.

Supplementary Video 2 An example video showing rasters of unit activity from six cells in anterior lateral motor cortex (ALM) (bottom) during prey capture behavior (a mouse chasing and capturing a cricket). Traces from the accelerometer channels are shown below. Spikes from cells 1, 2, 4, and 6 are plotted in real time (waveforms at top) and indicated in the audio by 50 ms tones of 800, 1,200, 1,400, and 1,600 Hz, respectively.

Supplementary Video 3 An example video of courtship behavior. A spectrogram (top) shows ultrasonic vocalizations as they occur during the video of the behavior shown below. Continuous LFP traces from 8 brain regions are shown below. Audio was pitch shifted in audacity to bring the vocalizations within the frequency range of human hearing. Note that this spatial arrangement of the cameras leaves ample room for mice to utilize the nosepoke ports of the interaction barrier.

Supplementary Video 4 Three example videos showing several epochs of natural cyclotorsion of the eye using the three different compositions of headset components shown in Figure 4 (with/without collimating lens, epi-illumination, and back-illumination). Optical conditions are listed before each example. Playback speed was 0.25x.

Supplementary Video 5 An example video using this system with infrared back-illumination pupillometry (iBip), which reveals the fundus and optic disc as the eye moves its position within the orbit of an awake freely-moving mouse. A cropped portion of the raw deinterlaced video (left) is shown with its inverse image (right) for clearer visualization of the fundus. A whisker between the camera and the eye happens to move in and out of frame throughout the video. The full-frame (uncropped) inverse image video follows at the end of the video. See Supplementary Video 4 for an iBip example showing the whiskers in white light, and Supplementary Video 6 for examples of iBip across sleep-wake cycles in the dark.

Supplementary Video 6 Example videos showing behavior across the sleep-wake cycle of an unrestrained mouse in the nest of the homecage using this system with iBip. Wake is shown in real-time, and is characterized by a large pupil diameter, and active movements of the eyes, whiskers, and limbs. NREM sleep is shown at 20x speed, and is characterized by immotility and slow large amplitude fluctuations of the pupil. Putative REM sleep is shown in real-time, and is characterized by a small pupil diameter and active twitching of the eyes, whiskers, and mystacial pad occurring during a background of immobility.

Supplementary Video 7 An example video showing the pupil and whiskers during the natural jumping behavior of a freely-moving mouse. Using the adjustable mounting system, different positions can be tested for pilot experiments without having to iteratively rebuild headsets (compare the different camera angles in Supplementary Videos 2, 7). This video also shows an example of the video signal obtained without an integrated IR LED (note how the illumination varies as the mouse moves relative to the overhead lighting).

### Appendices

Appendix 0 Parts list.

Appendix 1 Removal of IR filter.

Appendix 2 Clearing excess rubber adhesive.

Appendix 3 Stripping the camera module leads.

Appendix 4 Preparing wire segments for soldering onto the cameras.

Appendix 5 Soldering the wires onto the cameras and insulating them with silicone glue.

Appendix 6 Shielding, bending, weaving, and stripping the wires.

Appendix 7 Cementing the cameras onto the carrier.

Appendix 8 Adding the LEDs.

Appendix 9 Soldering the power and ground tethers.

Appendix 10 Shielding the weaving lattice and initial segment of the tethers.

Appendix 11 Attaching the headset to a headstage.

Appendix 12 Grounding the shields.
