# Supplementary material for: A Head-Mounted Multi-Camera System for Electrophysiology and Behavior in Freely-Moving Mice
Source: Front Neurosci. 2021 Jan 18;14:592417. doi: 10.3389/fnins.2020.592417 (PMC7874224; doi:10.3389/fnins.2020.592417)
Supplement: Appendix 0 — Parts list. [file Data_Sheet_1.PDF]

Appendices:

Appendix 0: Parts List

Part Number	Name	Source/Manufacturer	Part number or URL
1	LeeChatWin Analog Camera RS-306	AliExpress	link
2	Fusion 360	Autodesk	link
3	Cura	Ultimaker	link
4	Maker Select Plus 3D Printer	Monoprice	15711
5	Camera carrier	3D-print stl file	link
6	Video ground loop isolators	NewEgg	link
7	Loctite super glue	Loctite	1363589
8	30AWG Wrapping wire	NewEgg	link
9	Resistors	Amazon	link
10	Shrink tubing	Amazon	link
11	12 mm focal-length collimating lens	Lilly electronics	link
12	Fisher tape	Fisher Scientific	link
13	Micropore Surgical Tape	3M	70200412230
14	20G stainless-steel wire	Amazon	link
15	3 mm IR LED	Chanzon	100F3T-IR-FS-940NM
16	USB 2.0 Video Capture Card	UCEC	8541594679
17	Amplifier	Intan Technologies	#C3324 RHD 32ch + accel
18	RHD USB Interface Board	Intan Technologies	link
19	OpenEphys software		OpenEphys
20	Kilosort		Kilosort
21	Bonsai software		link
22	Webcam	ELP	ELP-USBFHD01M-BFV
23	IR dome USB camera	ELP	ELP-USBFHD05MT-DL36
24	Microphone	Brüel and Kjær	TYPE 4939-A-011
25	Lynx 22 sound card	Lynx	link

26	Audacity	Audacity	Audacity
27	Implant base	3D-print stl file	link
28	00-80	U-Turn Fasteners	link
29	18-8 washer	Amazon	B000FN1718
30	Teflon-coated stainless-steel wire	A&M Systems	791400
31	Small EIB pins	Neuralynx	link
32	EIB-36-PTB	Neuralynx	link
33	Tungsten wire (0.0007, Tungsten 99.95%, HFV insulation)	California Fine Wire Company	link
34	RTV silicone glue	Permatex	80050
35	EIB screws	Amazon	AMS90/8
36	Social cap	3D-print stl file	link
37	Skull screw	Grainger	2AE98
38	48 LED IR light source	Amazon	link
39	6000K LED Floodlight	Quans	Amazon
40	Clear plexiglass acrylic	ePlastics	ACRYCLR0.080FM48X72ACRYCLR0.080FM48X72
41	5500K 95+CRI light bulb	Litebox	HD-105
42	Adjustable IR LED light source	OSRAM	940nm LED Engin
43	Aluminum tape	Amazon	CECOMINOD062708
44	PTFE tape	Anti-Seize Technology	16030
45	Silver shielding paint	MG chemicals	842AR-15mL
46	Large EIB pins	Neuralynx	link
47	Electric connectors	Mill-Max	853-43-100-10-001000

Appendix 1: Removal of IR filter

Begin by unscrewing the lens located on the front of the camera (A). With the lens removed (B), place the camera out of the way and facedown on a clean surface to avoid any unwanted particles from accumulating on the sensor. Flip the lens over and begin using a scalpel blade around the edges of the filter to gently separate it from the lens. Once the filter has been sufficiently loosened around the edges, the blade will slide underneath it (C). Take care to avoid scratching the lens underneath during this process. Continue to work the blade around all sides of the filter until it can be fully removed from the lens (D). With the filter removed, screw the lens back onto the camera.

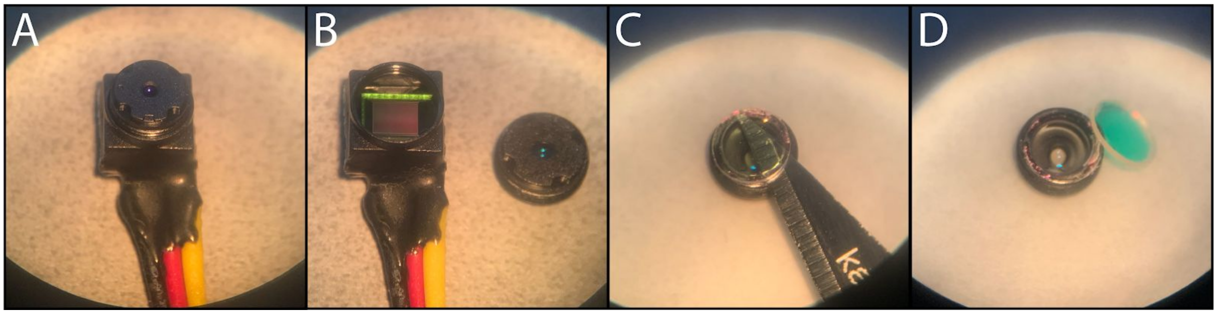

Test the video signal again using the camera's power and signal connectors, to confirm that the signal is now sensitive to an infrared light source and that the lens and sensor are free of any debris or damage that affects the video signal.

Appendix 2: Clearing excess rubber adhesive

The cameras often come with a small amount of external rubber adhesive (an example is shown in A), which can interfere with positioning when placed into a camera carrier. To prevent irregular placement, we recommend that you ensure that the four sides of the camera are clear of any rubber adhesive around the square-shaped core of the module. Gently remove adhesive using a scalpel and forceps until all sides of the camera are clear (as in B).

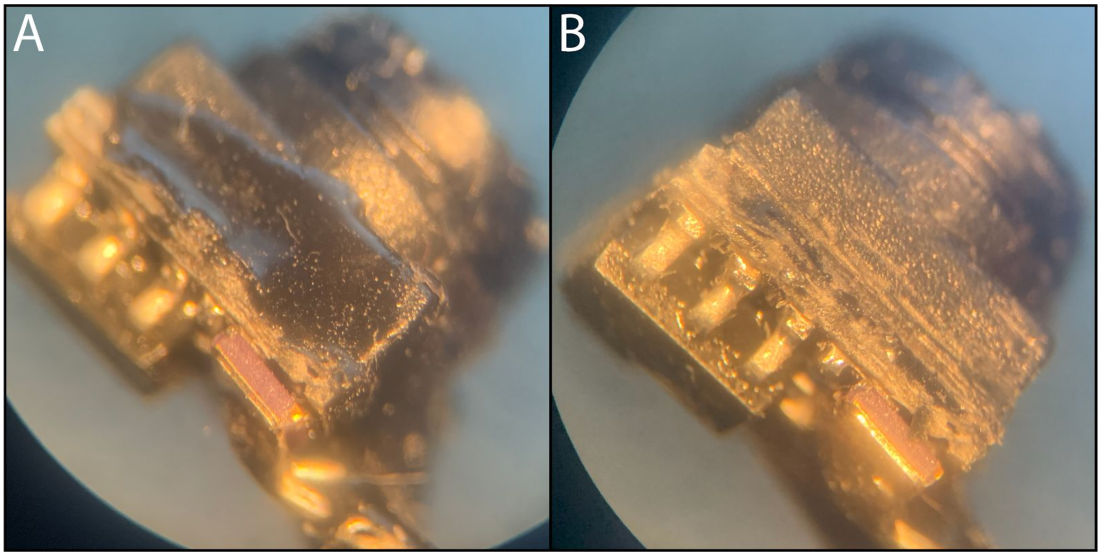

Appendix 3: Stripping the camera module leads

Use wire cutters to cut the camera module’s signal, power, and ground wires at least 5 mm from the camera modules. Then, using a scalpel blade and forceps, carefully strip the signal, power, and ground wires to the base of the camera module (B). Take care when stripping near the base of the module, and leave some excess rubber around their connections, so they are less likely to break from stress during assembly.

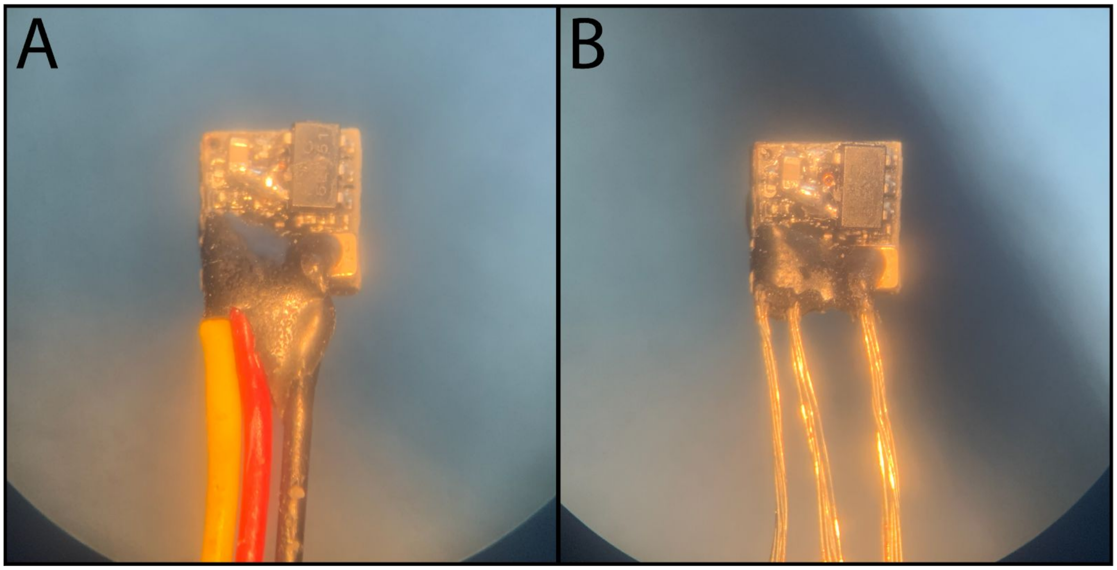

Appendix 4: Preparing wire segments for soldering onto the cameras

Place the cameras in their appropriate holder on a carrier, with the accompanying LED(s) in place to use for reference while you prepare the wires that will power them. Strip and cut wires to the appropriate lengths, and solder on resistors where necessary (e.g. 390 **Ω**, Part #9). Panel A shows an example of power and ground wires customized for a four-camera headset, with specific gaps in insulation to solder to the cameras. Panel B shows an enlarged view of the power and ground wires for a right-ear camera. The signal wires for each camera (not pictured here) should be cut to be as long as your desired tether length.

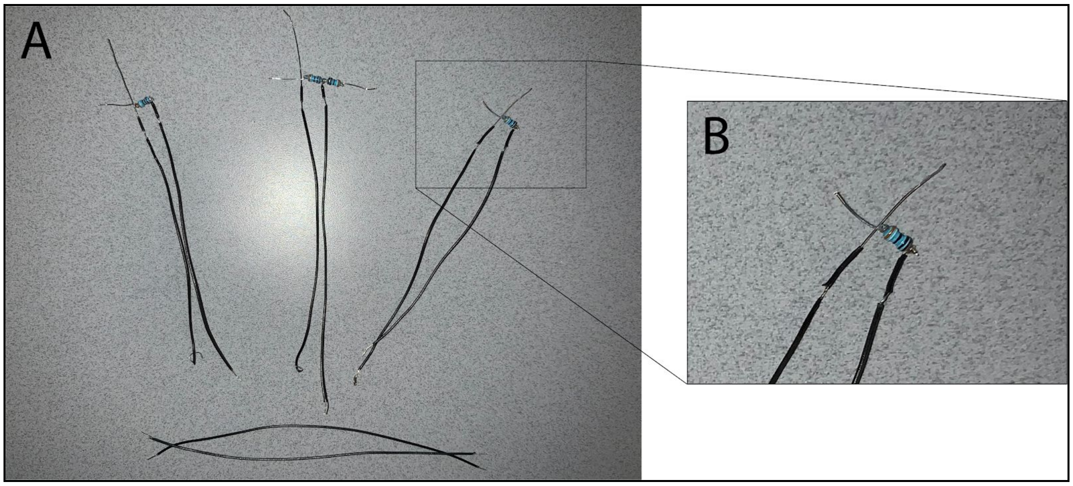

Appendix 5: Soldering the wires onto the cameras and insulating them with silicone glue

With a camera in its proper position on a carrier, solder the signal, power, and ground wires, so they are oriented in the proper direction towards the center of the carrier. This is shown for a left ear camera as an example in panels A, B, and C. Once soldered and trimmed, remove the camera from the carrier and coat the exposed wire with silicone glue (D) and allow it to dry, so they are fully insulated.

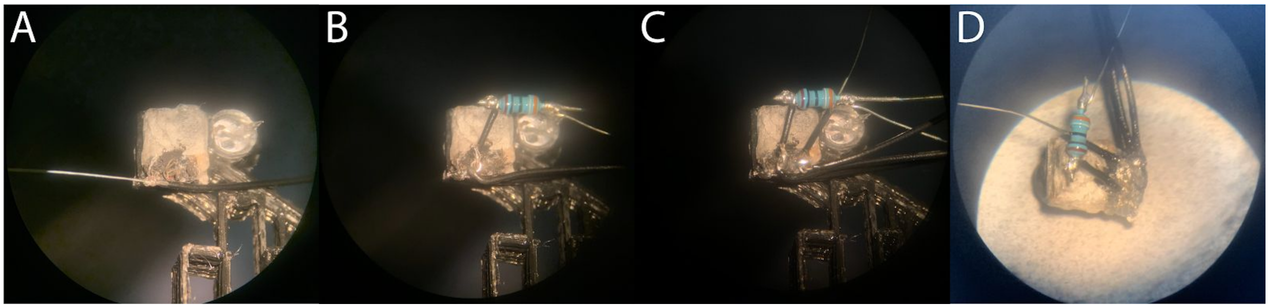

Appendix 6: Shielding, bending, weaving, and stripping the wires

Close proximity of the wires and cameras to the headstage can introduce noise into the electrode channels. For this reason, we used aluminum tape to shield the electrical components of the headset. Cut a section of aluminum tape (Part #43) with a 5 mm wide strip running about 25 mm in length off of one side (A). Peel the backing from the strip, make two small cuts, and reinforce the joint with a small piece of tape or superglue to prevent tearing (A). Place the camera module at this joint, with the wires running on top of the strip (B). Wrap the strip around the three wires (C). Use a multimeter at this point to confirm that the aluminum is not electrically connected to any of the wires. Place the camera module in its proper position on a carrier and bend the wrapped wires at the proper locations so they feed towards the weaving lattice at the center of the carrier (D). Mark the location on the aluminum where the wires meet the first lattice crossbar on the carrier (shown by the red arrow in D). Remove the camera from the carrier, and peel back the aluminum strip to your marked location. Place the camera back on the carrier, and route the ends of the power and ground wires down through separate lattice windows of your choosing, and mark the location where they emerge from the bottom. It's recommended to route the power wires in separate windows from the ground wires so they are well-separated when they are eventually soldered to the tethers as described in Appendix 9. Remove the camera from the carrier, and strip the ends of the power and ground wires to where you made your mark. Repeat this for each camera while it is in its proper position.

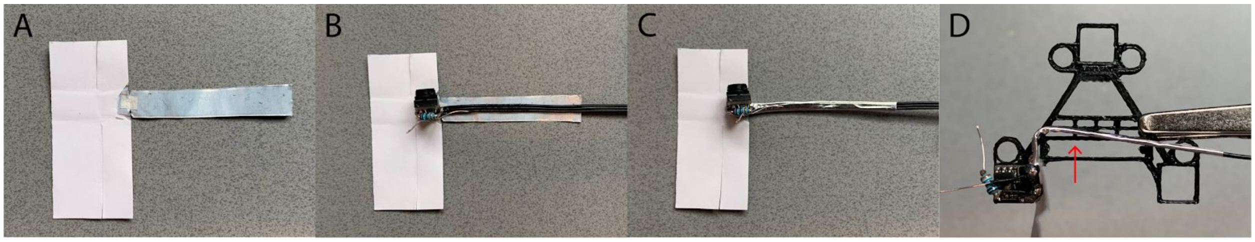

Appendix 7: Cementing the cameras onto the carrier

Place the camera on the carrier with the power and ground wires in their proper windows, and weave the camera's signal tether down through a lattice window, and back up, so it emerges upward from the center of the carrier (A). Slightly remove the camera from its holder, apply a small amount of superglue gel around the sides, and place it back in its holder, ensuring that the surface of the camera is perfectly flush with the bottom surface of the camera carrier (B). Once the glue has completely cured and the camera is solidified into place, peel off the paper from the unapplied aluminum tape, and wrap the camera module so it is as completely encased as possible, adding silicone glue or PTFE tape (Part #44) between the aluminum and camera components for insulation as needed, to ensure they don't become electrically connected. Confirm this using a multimeter. Repeat these steps for the remaining cameras.

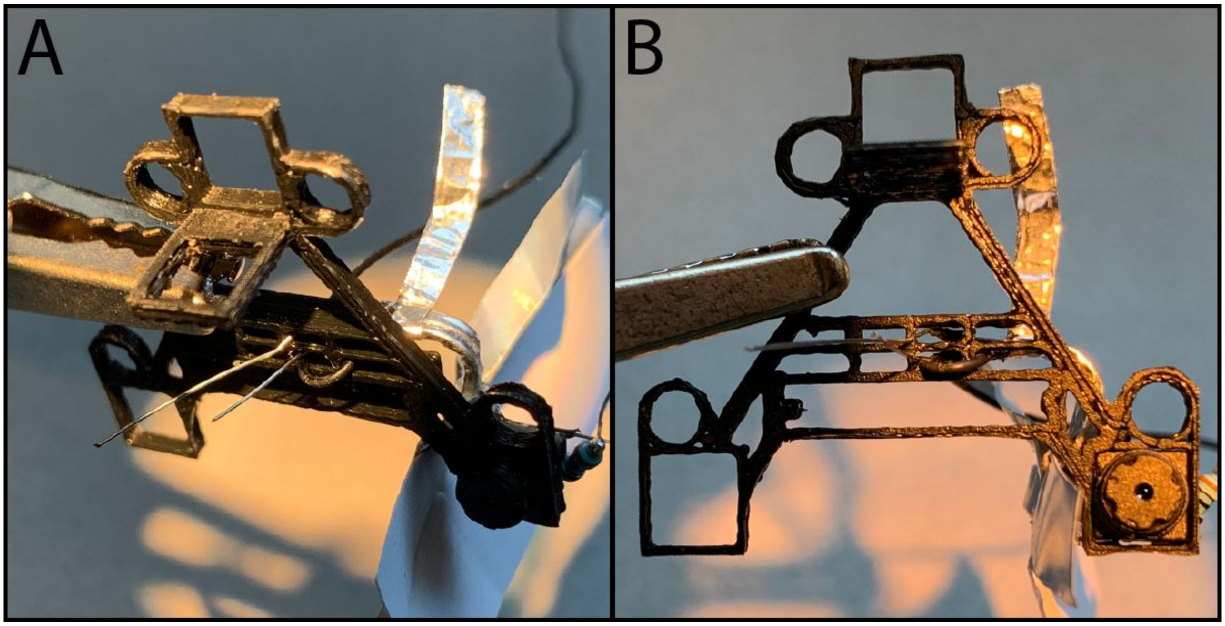

Appendix 8: Adding the LEDs

Trim the power and ground terminals of an IR LED, and insert it into an LED receiver on the carrier (shown by the black arrow in A). Wrap the local power and ground wires from the camera module to the leads of the LED (shown by the red and black arrows in B). Use a multimeter to ensure that no undesired connections to the shielding exist, and then solder them in place for a stable and permanent connection. Repeat these steps for the remaining LEDs.

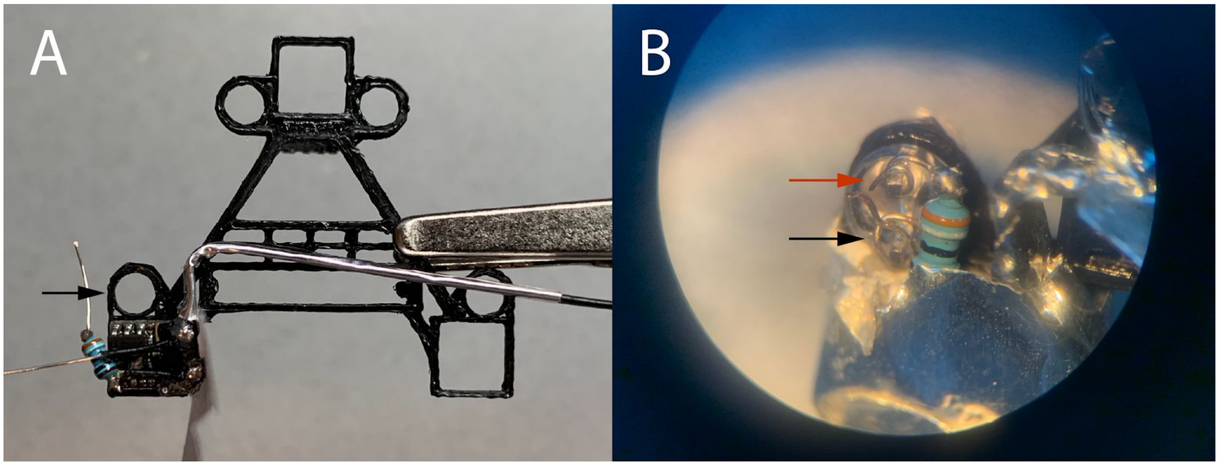

Appendix 9: Soldering the power and ground tethers

With all the cameras cemented in place and their power and ground wires extending from the bottom of the carrier (A), prepare two wires cut to your desired tether length to serve as the common power and ground tethers. Strip the ends of the wire, and feed it down through the appropriate window for the power or ground. Twist the 4 power (or ground) wires around the respective tether wire (B). Ensure that there is no connection between the power and ground wires using a multimeter. At this point, you should power the cameras to test that they all produce a proper video signal before moving forward. Solder the wrapped wires for a permanent connection, and completely cover this bare wire and solder with insulation so they can be wrapped with aluminum shielding in the next step.

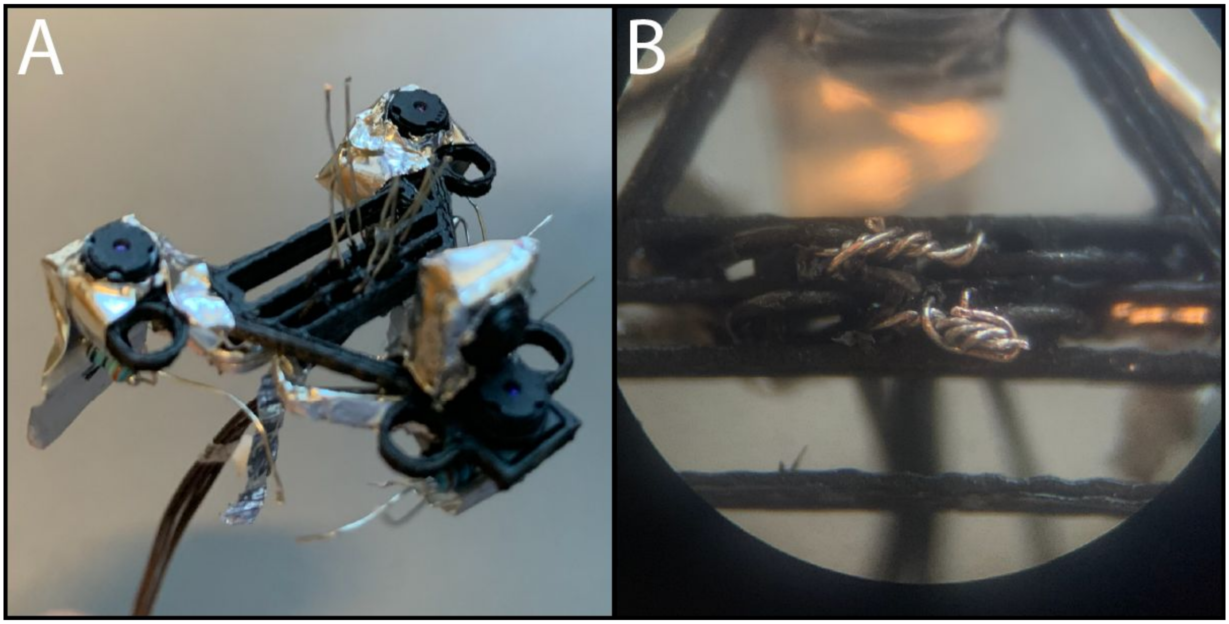

Appendix 10: Shielding the weaving lattice and initial segment of the tethers

Cut a 75x15 mm piece of aluminum tape, with 2x1 mm notches on each side about 11 mm from the top (A). Peel and remove the backing from the top to the point of the notches (B). Replace the backing onto the aluminum, and fold the top portion as shown (B). For clarity, the next steps are shown with an empty carrier. Insert the top portion through the front opening, from the underside of the carrier (C). Remove the protective paper (D). Insert the remaining end through the front portion of the headstage window (E). Pull both sides upwards, so they surround the weaving lattice as shown (F). Press them together, so they encase the woven wires (G) and tethers (not shown). Trim and fold the remaining tape so the first 4cm of the tethers are completely encased, shown here on a prepared carrier (H). All shielding combined, as shown in (H), weighed 84 mg.

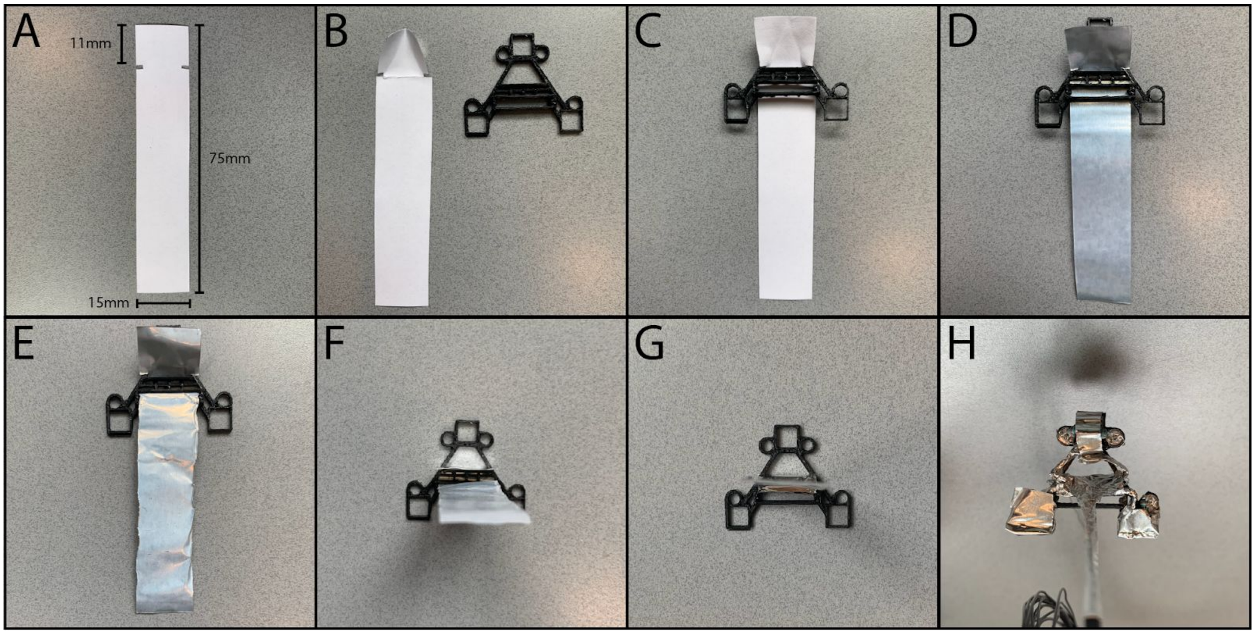

Appendix 11: Attaching the headset to a headstage

Unplug a headstage, and slide it up through the back of the headstage window on the headset with the front of the headstage facing forward, as seen with an empty carrier as an example in A. The fit is very tight, so we recommend practicing this on an empty carrier first to get an idea of how much force you'll need to use. Lower the headset until it reaches the bottom of the headstage as shown in B. Once placed on a headstage, the headset can be removed and reattached if necessary, but may cause wear and tear to the headset, and should be avoided.

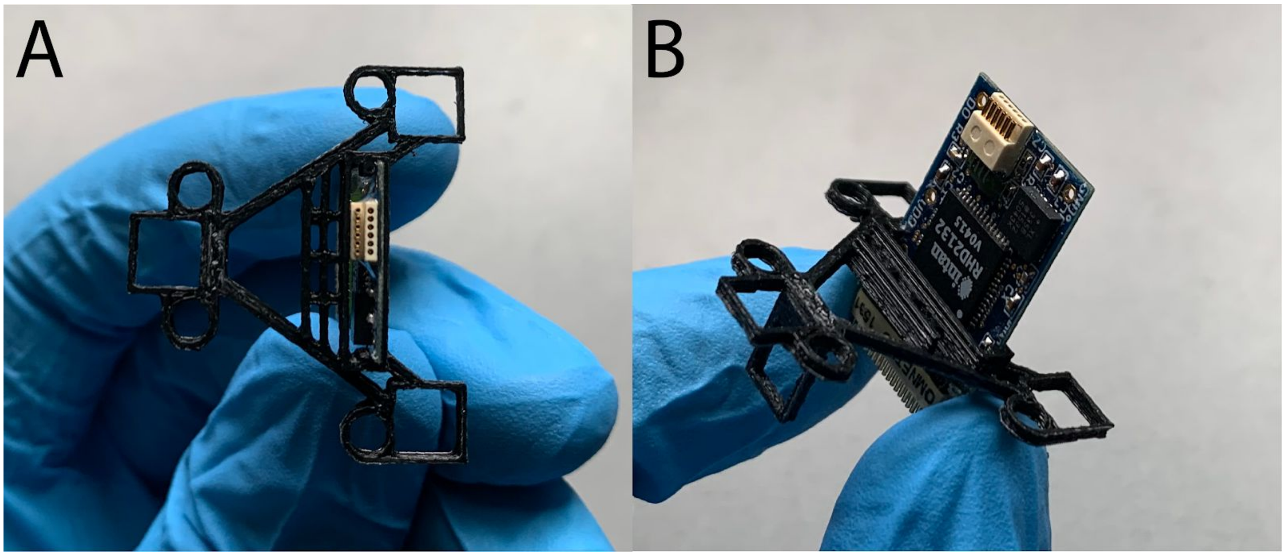

Appendix 12: Grounding the shields

Prepare stripped wire to serve as the grounding connections for the shields. Create a small puncture in each of the aluminum shields of the headset, and weave the wire through them to create an electrical connection. Use a multimeter to confirm that all pieces of shielding have a low resistance connection to the remaining free end of the shielding wire. Use the multimeter once again to confirm that none of the shields have an electrical connection to any camera components at this point as well. Use silver conductive paint (Part #45) to adhere the shielding wire to the shielding tape at each connection point. Pin the free end of the shielding wire to either the GND or REF of the headstage with a large gold pin (Part #46), and coat it in silicone glue for a stable, yet removable connection. If pinning to REF, you'll need to shorten the length of the pin, and pin from the front of the headstage so it can make a solid connection without hitting the back of the carrier.
